# 

**Published:** 1862-04

**Authors:** 


New Series, Vol. 5, No. 4. Whole Series, Vol. 19, No. 4.

### THE CHICAGO
### MEDICAL JOURNAL.

DANIEL BRAINARD, M. D.,
J. ADAMS ALLEN, M. D.,
Editors and Proprietors.

APRIL, 1862.

CHICAGO:

WM. PIGOTT, BOOK AND JOB PRINTER, 130 CLARK STREET.

1862.

